# The Impact of Inadequate Exposure to Epidermal Growth Factor Receptor–Tyrosine Kinase Inhibitors on the Development of Resistance in Non-Small-Cell Lung Cancer Cells

**DOI:** 10.3390/ijms25094844

**Published:** 2024-04-29

**Authors:** Daniela Frezzetti, Vincenza Caridi, Laura Marra, Rosa Camerlingo, Amelia D’Alessio, Francesco Russo, Serena Dotolo, Anna Maria Rachiglio, Riziero Esposito Abate, Marianna Gallo, Monica Rosaria Maiello, Alessandro Morabito, Nicola Normanno, Antonella De Luca

**Affiliations:** 1 Cell Biology and Biotherapy Unit, Istituto Nazionale Tumori-IRCCS-Fondazione G. Pascale, 80131 Naples, Italy; d.frezzetti@istitutotumori.na.it (D.F.); vincenza.caridi@istitutotumori.na.it (V.C.); laura.marra@istitutotumori.na.it (L.M.); r.camerlingo@istitutotumori.na.it (R.C.); serena.dotolo@istitutotumori.na.it (S.D.); am.rachiglio@istitutotumori.na.it (A.M.R.); r.espositoabate@istitutotumori.na.it (R.E.A.); marianna.gallo@istitutotumori.na.it (M.G.); m.maiello@istitutotumori.na.it (M.R.M.); a.deluca@istitutotumori.na.it (A.D.L.); 2 Laboratory of Toxicology Analysis, Department for the Treatment of Addictions, ASL Salerno, 84124 Salerno, Italy; al.dalessio@aslsalerno.it; 3 Institute of Endocrinology and Experimental Oncology, National Research Council of Italy, 80131 Naples, Italy; f.russo@ieos.cnr.it; 4 Thoracic Department, Istituto Nazionale Tumori-IRCCS-Fondazione G. Pascale, 80131 Naples, Italy; a.morabito@istitutotumori.na.it

**Keywords:** non-small-cell lung cancer, EGFR mutations, resistance, EGFR–tyrosine kinase inhibitors

## Abstract

Epidermal growth factor receptor (EGFR)-mutant non-small-cell lung cancer (NSCLC) patients treated with EGFR–tyrosine kinase inhibitors (TKIs) inevitably develop resistance through several biological mechanisms. However, little is known on the molecular mechanisms underlying acquired resistance to suboptimal EGFR-TKI doses, due to pharmacodynamics leading to inadequate drug exposure. To evaluate the effects of suboptimal EGFR-TKI exposure on resistance in NSCLC, we obtained HCC827 and PC9 cell lines resistant to suboptimal fixed and intermittent doses of gefitinib and compared them to cells exposed to higher doses of the drug. We analyzed the differences in terms of EGFR signaling activation and the expression of epithelial–mesenchymal transition (EMT) markers, whole transcriptomes byRNA sequencing, and cell motility. We observed that the exposure to low doses of gefitinib more frequently induced a partial EMT associated with an induced migratory ability, and an enhanced transcription of cancer stem cell markers, particularly in the HCC827 gefitinib-resistant cells. Finally, the HCC827 gefitinib-resistant cells showed increased secretion of the EMT inducer transforming growth factor (TGF)-β1, whose inhibition was able to partially restore gefitinib sensitivity. These data provide evidence that different levels of exposure to EGFR-TKIs in tumor masses might promote different mechanisms of acquired resistance.

## 1. Introduction

Epidermal growth factor receptor (EGFR)–tyrosine kinase inhibitors (TKIs) such as gefitinib, afatinib, and osimertinib represent the standard of care for the treatment of advanced non-small-cell lung cancer (NSCLC) patients carrying EGFR-activating mutations. Robust clinical evidence has shown the superiority of such agents to conventional platinum-based chemotherapy in terms of progression-free survival and response rates [1]. However, the clinical efficacy of EGFR-TKIs is limited by the emergence of resistance, which results in disease progression in most if not all cases [1,2].

Acquired resistance to EGFR-TKIs in NSCLC patients can develop through either biological or pharmacological mechanisms. Biological resistance is acquired consequently to the clonal evolution of cancer cells under the pressure of treatments administered at optimal doses. In this regard, genomic alterations such as the EGFR T790M mutation; the activation of bypass signaling pathways, including RAS/ERK, PIK3/AKT, and MET alterations; the histological transformation into small-cell lung cancer (SCLC); and the acquisition of the epithelial–mesenchymal transition (EMT) phenotype are the main mechanisms responsible for biological resistance to EGFR-TKIs [3,4]. Pharmacological resistance occurs when cancer cells theoretically retain intrinsic drug sensitivity, but the concentration of EGFR-TKIs at the target tumor sites is not sufficient to obtain an adequate pharmacological response [5]. Biological resistance and pharmacological resistance are likely correlated. In fact, an insufficient and/or intermittent drug exposure might favor the development of molecular mechanisms responsible for acquired resistance in cancer cells [5].

Although biological mechanisms of acquired resistance to EGFR-TKIs have been extensively explored in several in vitro models, the molecular mechanisms involved in the resistance of NSCLC cells exposed to suboptimal/intermittent doses of EGFR-TKIs have not been fully elucidated yet. In this regard, an insufficient and/or heterogeneous exposure of cancer cells to effective EGFR-TKI levels might promote the emergence of resistance with molecular mechanisms different from those induced when cancer cells are exposed to optimal doses of the drug. Starting from this observation, we investigated whether different modalities of drug exposure influenced the acquired resistance to EGFR-TKIs, using two EGFR-mutant NSCLC cell lines exposed to continuous or intermittent suboptimal doses of gefitinib until the acquisition of resistance. By using this approach, we demonstrated that both the modality of drug exposure and the cellular genetic background might significantly affect the mechanisms of biological resistance to EGFR-TKIs. Our results might provide the rationale for the development of novel therapeutic strategies for overcoming resistance and possibly improving the efficacy of EGFR-TKIs in EGFR-mutant NSCLC patients.

## 2. Results

### 2.1. Generation and Characterization of Gefitinib-Resistant (GR) Non-Small-Cell Lung Cancer (NSCLC) Cell Lines

**Figure 1:**
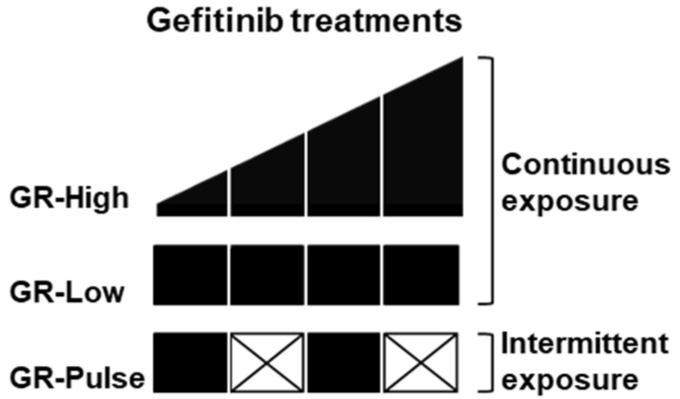
Schedule of gefitinib treatment of non-small-cell lung cancer (NSCLC) cells. Schematic representation of the different modalities of exposure with high or low concentrations of gefitinib used over a period of 6 months to obtain the different gefitinib-resistant (GR) cell lines. Incremental and low treatments with gefitinib are represented with black blocks. White crossed blocks represent treatments with gefitinib-free medium.

To investigate the molecular mechanisms involved in the resistance of NSCLC cells exposed to suboptimal doses of EGFR-TKIs, we treated two NSCLC cell lines carrying an EGFR exon 19 deletion (HCC827 and PC9) with low doses of gefitinib, using two drug regimens, continuous and intermittent, to obtain gefitinib-resistant (GR)-Low and GR-Pulse cells, respectively. At the same time, we treated HCC827 and PC9 cells with increasing concentrations of gefitinib to obtain two cell lines resistant to high doses of the drug, namely HCC827 GR-High and PC9 GR-High (Figure 1). After 6 months of drug exposure, the GR cells were able to routinely grow in gefitinib-containing medium at the different concentrations used for the acquisition of the resistance.

In agreement with previous data [6,7], the HCC827 and PC9 parental cell lines were highly sensitive to gefitinib, with IC50 values of 13.06 nM and 77.26 nM, respectively (Table 1). In contrast, the GR cells were resistant to high concentrations of gefitinib, showing IC50 values > 4 µM.

The kinetics of growth for the GR cell lines were similar to those of their respective parental cells, demonstrating that the resistant cells did not gain a proliferative advantage over their parental cell lines (Figure S1).

Genomic profiling of the GR cells was performed with a targeted next-generation sequencing (NGS)-based panel covering relevant molecular alterations in 161 cancer genes (Table S1). We did not detect the EGFR T790M mutation or MET amplification, recurrent mechanisms of acquired resistance to gefitinib, in any of the GR cell lines.

In agreement with previous studies [7,8], the NGS analysis revealed a progressive decrease in the EGFR exon 19 deletion and in the EGFR amplification through the passage during selection only in the HCC827 GR-High cell line, but not in the GR-Low or GR-Pulse cells.

### 2.2. Differential Activation of EGFR Signaling in GR NSCLC Cells

**Figure 2:**
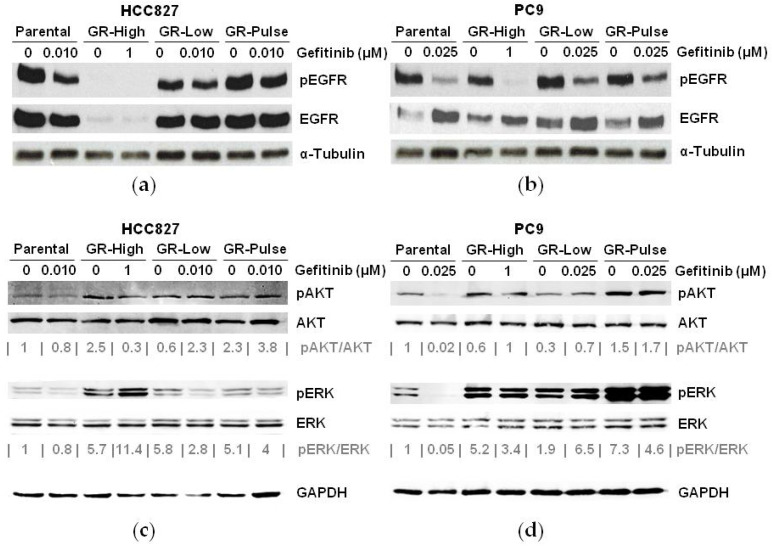
Aberrant activation of EGFR signaling in GR cells. (**a**,**b**) Western blot analysis of EGFR expression and activation levels in HCC827 (**a**) and PC9 (**b**) parental and GR cells, in the absence or presence of gefitinib at the indicated concentrations. An anti-α-tubulin antibody was used for normalization. (**c**,**d**) Western blot analysis of expression and activation levels of ERK and AKT in HCC827 (**c**) and PC9 (**d**) parental and GR cells, in the absence or presence of gefitinib at the indicated concentrations. An anti-GAPDH antibody was used for normalization. Densitometric value ratios were calculated for phospho-ERK/total ERK (pERK/ERK) and phospho-AKT/total AKT (pAKT/AKT).

We next analyzed the effects of gefitinib on EGFR signaling in GR cells using Western blot analyses. The GR cells were exposed to the same concentrations of gefitinib as those in which they were routinely maintained. Parental cells were treated with the doses of gefitinib used for the GR-Low and GR-Pulse cells, corresponding to approximately 35% growth inhibition. The HCC827 GR-Low and GR-Pulse cells retained their EGFR protein expression, whereas in the HCC827 GR-High cells, an almost complete suppression of the EGFR protein expression was observed (Figure 2a), in agreement with the loss of EGFR amplification and in line with previous reports [7]. The EGFR expression was gradually lost until disappearance in HCC827 GR-High during the selection, as previously described [7]. Treatment with gefitinib induced a reduction in phospho-EGFR levels that was more significant in the HCC827 parental cells when compared with the HCC827 GR-Low and GR-Pulse cells (Figure 2a). EGFR activation was significantly reduced upon gefitinib treatment in the parental and PC9 GR cell lines, which showed no alteration in the total levels of EGFR protein expression (Figure 2b). The analysis of signaling pathways downstream of the EGFR confirmed that gefitinib treatment reduced AKT and ERK phosphorylation in both the HCC827 and PC9 parental cells (Figure 2c,d). The HCC827 GR-High and GR-Pulse cells had higher basal levels of phospho-AKT than the HCC827 parental and GR-Low cells, whereas all HCC827 GR cells showed increased basal levels of phospho-ERK than the parental cells (Figure 2c). Interestingly, gefitinib treatment induced an increased activation of phospho-ERK in the HCC827 GR-High cells, whereas a persistent activation of AKT was observed in the HCC827 GR-Low and GR-Pulse cells, suggesting different dynamics of intracellular signaling pathways in these different clones (Figure 2c). In the PC9 GR cells, we found a persistent activation of AKT following gefitinib treatment in all cell lines, and only a slight reduction in the levels of ERK activation, whose basal levels were higher in the resistant cells when compared to the parental cells (Figure 2d).

Collectively, our data indicated that the aberrant activation of EGFR downstream signaling might contribute to the acquired resistance in GR cell lines exposed to suboptimal doses of EGFR-TKIs.

### 2.3. Analysis of the Transcriptomic Profiles of the GR NSCLC Cell Lines

**Figure 3:**
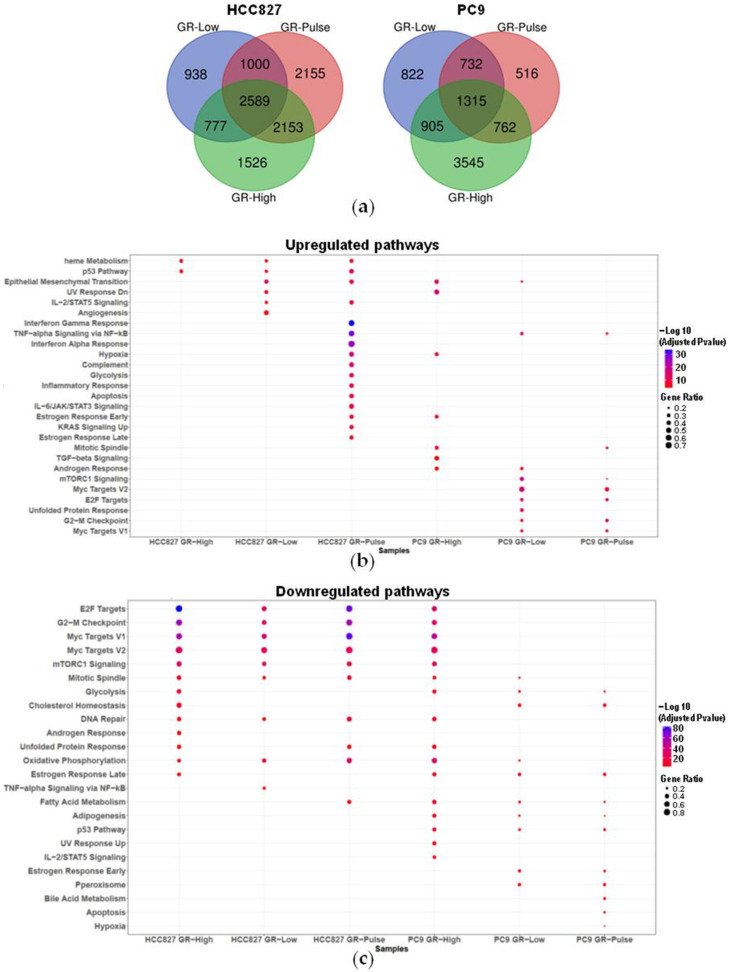
RNA-seq analysis. (**a**) Venn diagram showing the number of common and unique DEGs in GR cell lines compared to their respective parental cells. (**b**,**c**) Dot plots showing significantly enriched pathways affected by upregulated (**b**) and downregulated (**c**) DEGs in GR cells. The size of the dots is based on the fraction of DEGs included in each enriched pathway, whereas each dot’s color depends on the statistical significance of the enrichment, expressed as a −log10 adjusted *p*-value.

We next analyzed the transcriptomic profile of the different GR clones. Significant differences were observed between the transcriptomes of the parental and resistant cell lines (Figure S2). In the resistant cell lines, we identified a high number of differentially expressed genes (DEGs) with a log2 fold change ≠ 0 relative to the parental cells and an adjusted *p*-value < 0.05 (Figure S3 and Table S2). However, the fraction of DEGs in common between the resistant cell lines was quite low: 23.2% (2589/11,138 DEGs) for the HCC827 GR cell lines and 15.3% (1315/8597 DEGs) for the PC9 GR cell lines, suggesting a high heterogeneity in the transcriptomic profiles among the different resistant clones (Figure 3a).

A pathway enrichment analysis revealed that increased expressions of genes associated with the EMT occurred in the majority of the GR cells (HCC827 GR-Low and GR-Pulse cells and PC9 GR-High and GR-Low cells) (Figure 3b). Other pathways significantly upregulated in at least three of the GR cell lines included relevant oncogenic pathways such as the p53, TNF-alpha signaling, and heme metabolism pathways (Figure 3b). Among the significantly downregulated pathways, the mitotic spindle and oxidative phosphorylation pathways were found to be downregulated in all GR cell lines except the PC9 GR-Pulse cells (Figure 3c). Other pathways, including the E2F target, G2-M checkpoint, Myc target, mTORC1 signaling, DNA repair, and fatty acid metabolism pathways, were downregulated in at least four of the resistant cell lines (Figure 3c). Importantly, some pathways involved in tumorigenesis and cancer progression were specifically enriched only in cell lines resistant to suboptimal doses of gefitinib, such as angiogenesis in the HCC827 GR-Low cells and the inflammatory response pathway in the HCC827 GR-Pulse cells. Interestingly, only in the HCC827 GR-Pulse cells did we observe a significant upregulation of interferon-alpha and -gamma responses, which are associated with innate and adaptive immunity (Figure 3b). These data suggest that different pathways might be involved in the resistance to suboptimal doses of EGFR-TKIs and that their enrichment depends on both the cellular genetic background and the modality of gefitinib exposure.

**Figure 4:**
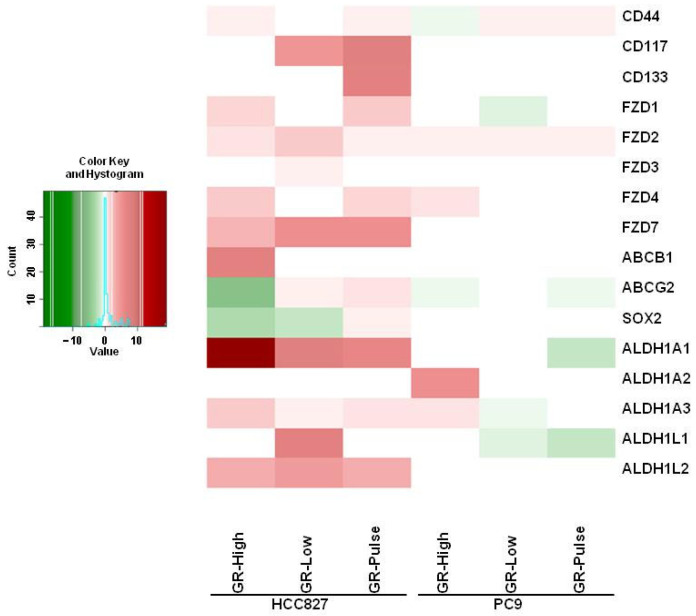
Cancer stem cell marker expression in GR cells. RNA-seq heatmap showing cancer stem cell marker expression in GR cells compared to their relative parental cells. Upregulated DEGs are shown in red, whereas downregulated DEGs are shown in green. Data are expressed as log2 fold changes versus the parental cells.

Finally, among the upregulated DEGs, we observed in the GR cells a strong induction of several cancer stem cell (CSC) markers previously identified in NSCLC [9]: a cluster of differentiation antigens such as CD117 and CD133, cell membrane transporters such as ABCB1 and ABCG2, Frizzled family receptor (FZD) members, and ALDH1 isoforms (Figure 4). Some CSC markers were upregulated in at least four GR cell lines, including CD44 and ALDH1A3. The increase in CSC markers was generally more pronounced in the HCC827 GR cells as compared with PC9. Interestingly, we found that CSC markers were induced only in the cells exposed to low doses of gefitinib, such as CD117 in the HCC827 GR-Low and GR-Pulse cell lines, CD133 in the HCC827 GR-Pulse cells, and ALDH1L1 in the HCC827 GR-Low cells (Figure 4).

### 2.4. Epithelial–Mesenchymal Transition and Migration in GR NSCLC Cells

The acquired resistance of NSCLC cells to EGFR-TKIs has been previously associated with the EMT [10]. As the expression of genes associated with the EMT was significantly enriched in both HCC827 and PC9 GR cells, we investigated whether the exposure to different doses of gefitinib induced mesenchymal features.

**Figure 5:**
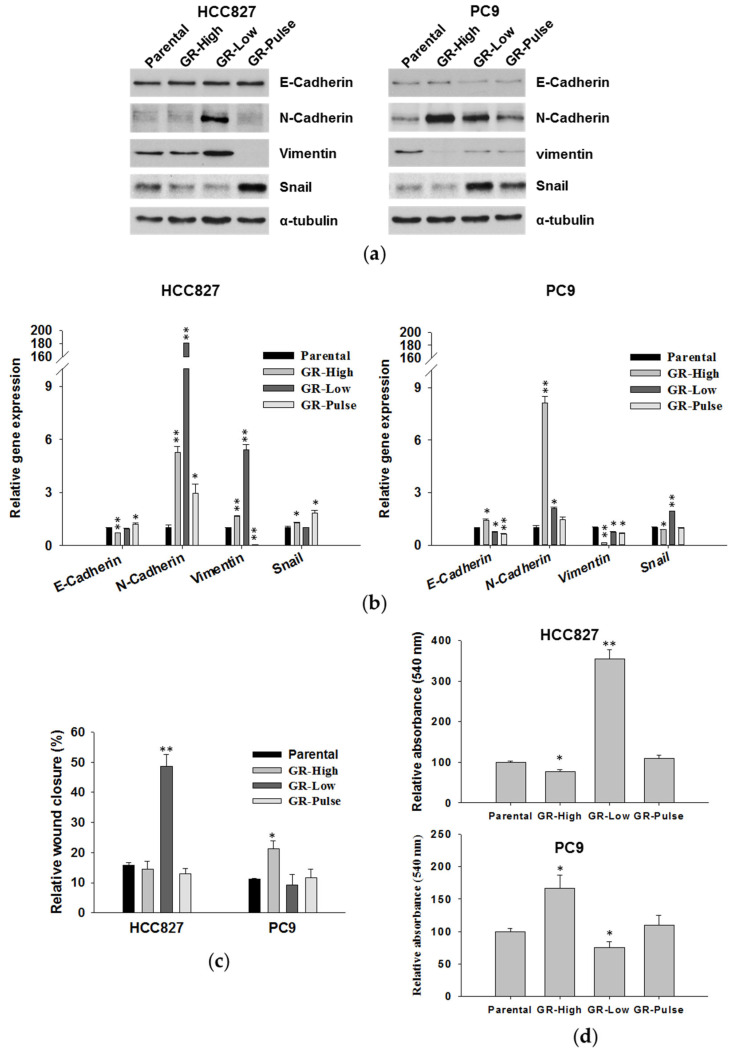
Analysis of EMT markers and migratory ability of GR cells. (**a**) Western blot analysis of E-cadherin, N-cadherin, Vimentin, and Snail expression in parental and GR cell lines. For normalization, the anti-α-tubulin antibody was used. (**b**) Real-time PCR analysis of EMT marker expression in GR cells compared to their respective parental cell lines. * *p* < 0.05 and ** *p* < 0.005 for comparison between GR versus parental cells (two-tailed Student’s *t*-test). (**c**) Analysis of wound-healing assays for parental and GR cells. * *p* < 0.05 and ** *p* < 0.005 for comparison between GR versus parental cells (two-tailed Student’s *t*-test). (**d**) Transwell migration assays for parental and GR cells. Cells were allowed to migrate through a polycarbonate membrane toward a medium containing FBS as a chemoattractant. * *p* < 0.05 and ** *p* < 0.005 for comparison between parental versus GR cells (two-tailed Student’s *t*-test).

Overall, a significant increase in the levels of the N-cadherin protein was observed in the HCC827 GR-Low cells and in all PC9 GR cells, compared to their relative parental cell lines (Figure 5a). No significant alterations in the levels of E-cadherin were observed in the GR cells compared to the parental cell lines, whereas a strong induction of vimentin expression was observed only in the HCC-827 GR-Low cells (Figure 5a). The levels of Snail were increased in the HCC827 GR-Pulse cells and in the PC9 GR-Low and GR-Pulse cell lines. The regulation of EMT markers in the GR cells was more evident at the transcriptional level, as confirmed by the strong induction of N-cadherin and vimentin mRNA expression levels in the HCC827 GR-Low cells and of N-cadherin mRNA expression in the PC9 GR-High cells compared to their respective parental cells (Figure 5b).

Since the acquisition of the EMT phenotype induces cancer cell motility [11], we next evaluated whether cell migration was increased in GR cells. In wound-healing assays, the HCC827 GR-Low cells showed a significant increase in migration compared to the parental cells and other GR cell lines, whereas a slightly increased migratory ability was observed in the PC9 GR-High cells (Figure 5c and Figure S4a). These data were confirmed in transwell migration assays, in which was observed an increase in cell migration of about 3.5-fold in HCC827 GR-Low and 1.6-fold in PC9 GR-High cells compared to the respective parental lines (Figure 5d).

### 2.5. TGF-β1 Expression in HCC827 GR Cell Lines

**Figure 6:**
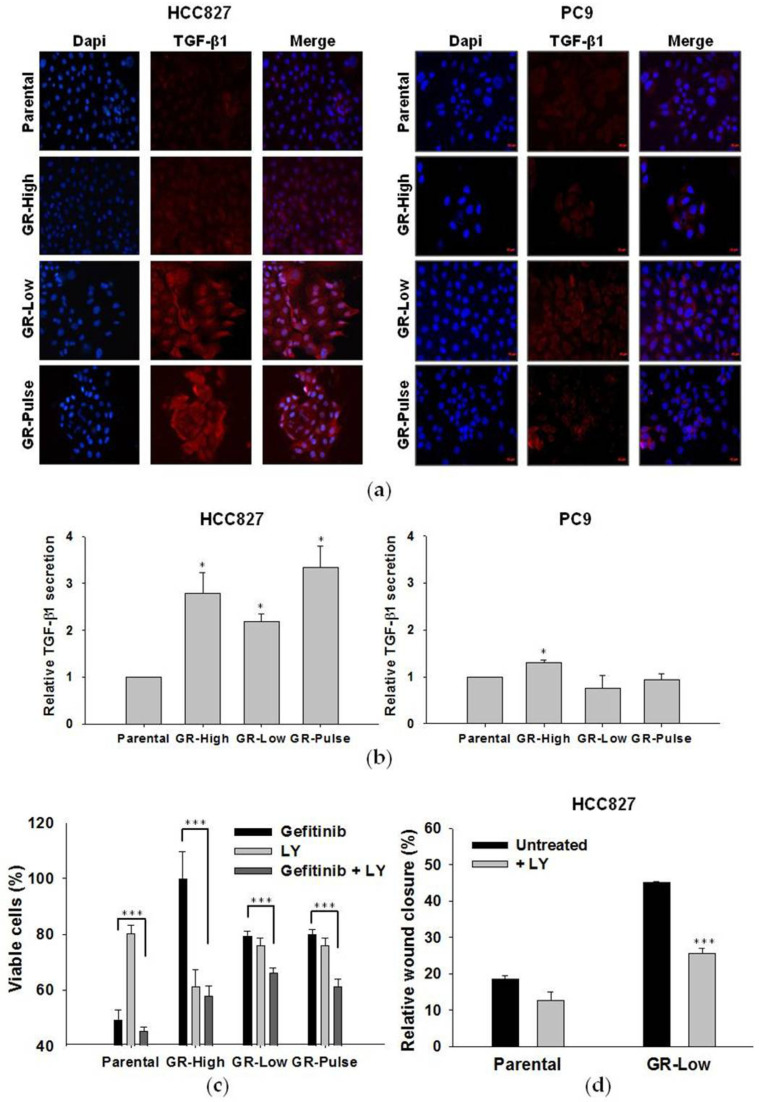
Evaluation of TGF-β1 expression in GR cells and its role in resistance and cell migration. (**a**) Immunofluorescence analysis of TGF-β1 expression (signaled in red) in HCC827 and PC9 parental and GR cell lines. Nuclei were stained with DAPI (signaled in blue). In the images, 20× magnification was used. (**b**) Immunoassay analysis of TGF-β1 levels in conditioned media from parental and GR cell lines. TGF-β1 levels were normalized for the cell number determined at the harvesting time and folds were calculated versus their respective parental cells. The values reported are the means from two independent experiments, each performed in duplicate. * *p* < 0.05 for comparison between parental versus GR cells (two-tailed Student’s *t*-test). (**c**) Cell proliferation assay on HCC827 parental and GR cells treated for 72 h with gefitinib (100 nM) and LY2109761 (LY, 5 µM), alone and in combination. *** *p* < 0.0005 for comparison between cells treated with gefitinib alone versus those treated with the drug combination (two-tailed Student’s *t*-test). (**d**) Analysis of wound-healing assays for HCC827 parental and GR-Low cells untreated and treated with LY (5 µM). *** *p* < 0.0005 for comparison between the treated versus untreated cells (two-tailed Student’s *t*-test).

As the transforming growth factor (TGF)-β1 is a strong inducer of the EMT [12,13] and a positive regulator of the CSC phenotype [14,15], and a TGB-β1-induced EMT has been previously correlated to EGFR-TKI resistance in NSCLC cells [16,17], we measured TGF-β1 expression in the GR cells. Through an immunofluorescence analysis, we observed that all HCC827 GR cells expressed higher levels of intracellular TGF-β1 compared to their parental cell line, whereas no significant differences in TGF-β1 expression were observed between the PC9 parental and GR cells (Figure 6a). Accordingly, all HCC827 GR cell lines and none of the PC9 GR cell lines showed increased levels of TGF-β1 secreted in conditioned media compared to their parental cells (Figure 6b). For these reasons, we focused our attention on the role of TGF-β1 in HCC827 cells.

Treatment with LY2109761 (LY), an inhibitor of the TGF-β receptor (TGFβR), partially rescued the sensitivity to gefitinib in HCC827 GR cell lines (Figure 6c) and significantly affected the increased migratory ability of the HCC827 GR-Low cells in wound-healing assays (Figure 6d and Figure S4b).

These data suggest that TGF-β1 induction regulates the EMT and migration in selected subtypes of NSCLC cells with acquired resistance to EGFR-TKIs, and that TGF-β1 signaling blockade might reduce resistance to gefitinib.

## 3. Discussion

It has been hypothesized that pharmacodynamic mechanisms reducing EGFR-TKI delivery to the target sites might constitute an important source of the variability of treatment responses among patients with NSCLC [5,18]. Indeed, dose reductions or interruptions, increased metabolism or reduced absorption, hypoperfusion, and a poor penetration of the blood–brain barrier may favor the development of pharmacological resistance to anti-EGFR agents in EGFR-addicted NSCLC patients [5,19]. In this regard, the combination of EGFR-TKIs with anti-angiogenic drugs that induce the normalization of tumor vasculature has improved the clinical outcome of EGFR-mutant NSCLC patients, possibly also through an enhanced delivery of anti-EGFR agents to the target tumor sites [20,21,22,23].

In this study, we tried to mimic the exposure to suboptimal concentrations of EGFR-TKIs by exposing EGFR-mutant NSCLC cells to low and intermittent doses of gefitinib. Interestingly, we observed significant differences in the resistant phenotypes of cells exposed to suboptimal gefitinib as compared to cells that developed resistance with the standard approach of increasing the concentration of the drug in a stepwise manner. In particular, the HCC827 cells resistant to high doses of gefitinib showed a complete suppression of EGFR protein expression and of EGFR gene amplification, in agreement with previous data [7]. In contrast, the HCC827 GR-Low and GR-Pulse cells carried levels of EGFR expression similar to their parental cells, demonstrating that the exposure to suboptimal or intermittent doses of gefitinib did not affect the expression of the EGFR, thus underlying the relevance of the modality of drug exposure for the mechanisms of acquired resistance. To our knowledge, the mechanisms involved in the resistance to EGFR-TKIs of NSCLC cells exposed to prolonged suboptimal concentrations of drugs have not been explored yet. Only one study has suggested that relatively low doses of erlotinib in HCC827 cells accelerated the emergence of resistance through the loss of EGFR amplification [8]. However, in this report, a different EGFR-TKI (erlotinib) and doses higher than the IC50 values were used, as compared with our strategy to employ low levels of the drug [8].

Collectively, our data are in agreement with previous studies suggesting that the loss of EGFR expression is a mechanism involved in the resistance to gefitinib of cancer cells exposed to optimal doses of gefitinib. Conversely, other mechanisms might be involved in the resistance of cells exposed to suboptimal doses of this drug.

Constitutive activation of both the AKT and/or ERK signaling pathways has been previously demonstrated to be involved in the acquired resistance to EGFR-TKIs. We did not find a consistent pattern of resistance among the different GR cell lines. We found a persistent activation of AKT in all GR cell lines, except for the HCC827 GR-High cell line, which showed a high level of ERK activation, suggesting that the expression of mutant EGFR is mandatory to sustain AKT signaling in this NSCLC cell line. However, we found a constitutive activation of ERK in all PC9 GR clones, indicating that the cellular genetic background significantly contributes to the activation of different EGFR downstream effectors in EGFR-TKI-resistant cells, despite our results confirming that the AKT and/or ERK signaling pathways are also persistently activated when tumor cells are exposed to suboptimal doses of gefitinib, similarly to cells exposed to higher escalating doses of the drug [7,8].

Although different studies have shown that transcriptomic changes occur in NSCLC cells after EGFR-TKI treatment, the complexity of resistance-induced transcriptional reprogramming is still far from being fully understood [24,25,26,27]. More importantly, to the best of our knowledge, no previous studies on the effects of prolonged exposure to suboptimal doses of EGFR-TKIs on the whole transcriptomes of NSCLC cells have been reported to date. Significant differences in the number and function of DEGs were observed between HCC827 and PC9 GR cell lines, also depending on the different schedules of treatment. Among the pathways that were significantly regulated by DEGs in the GR NSCLC cells, we found the EMT, p53, mTORC1, and oxidative phosphorylation signaling pathways, in agreement with previously reported RNA-seq data on EGFR-TKI-resistant NSCLC cell lines [24,27,28]. Intriguingly, we observed that suboptimal exposure to gefitinib significantly affected gene expression in a cell-type-specific manner, with several enriched pathways (the E2F target, G2-M checkpoint, Myc target, and mTORC1 signaling pathways) showing opposite regulation in HCC827 and PC9 cells (downregulated in the HCC827 GR-Low and GR-Pulse cells and upregulated in the PC9 GR-Low and GR-Pulse cells). Other pathways, such as the interferon-alpha and -gamma responses, were strongly upregulated only in the HCC827 GR-Pulse cells, suggesting that intermittent exposure to low doses of gefitinib might regulate specific pathways in drug-resistant NSCLC cells.

Most GR cell lines showed EMT features. An induced EMT has been correlated to an acquired resistance to EGFR-TKIs in NSCLC [11,29]. However, our data suggest that EMT induction depends on both drug exposure and genetic background. Indeed, the strongest expression of EMT markers was found in the HCC827 GR-Low and PC9 GR-High cells, although EMT markers were expressed to some extent in almost all resistant clones. The GR cells maintained their expression of the epithelial marker E-cadherin and acquired only some of the canonical EMT markers, suggesting the development of a hybrid epithelial/mesenchymal phenotype characterized by a co-expression of epithelial and mesenchymal markers. Partial EMTs have been described in several types of cancer, including lung cancer, and could promote resistance to anti-cancer drugs and enhance the invasiveness of cancer cells [30,31,32]. In fact, cancer cells with partial EMTs have been shown to have a higher metastatic potential compared to cells with only mesenchymal features [33,34,35]. Moreover, several studies have reported a significant correlation between the EMT and the acquisition of the CSC phenotype [36,37]. In particular, it has been reported that cancer cells with a hybrid EMT phenotype present more stem-like features compared to the original epithelial or fully mesenchymal states, thus reinforcing the correlation between partial EMTs and invasive/metastatic properties [38]. In this respect, we found that the exposure to either high or low levels of gefitinib induced the expression of CSC markers, although this phenomenon was more pronounced in the HCC827 cells. In future studies, we will perform experiments in three-dimensional models in order to investigate the molecular mechanisms involved in the EMT and the acquisition of the CSC phenotype in resistant clones, thus mimicking in vivo conditions [39]. Different mechanisms have been reported to be involved in EMT-associated resistance to EGFR-TKIs, such as the overexpression of AXL receptor tyrosine kinase [40], SMO gene amplification [41], the overexpression of C-X-C chemokine receptor 7 [42], the activation of yes-associated protein and forkhead box protein M1 [28], and increased TGF-β1 levels [16,17]. In particular, increased TGF-β1 secretion has been previously reported in NSCLC cell lines resistant to high doses of gefitinib, as well as in HCC827 cells resistant to erlotinib and osimertinib [16,17]. Moreover, a prolonged exposure of NSCLC cells to TGF-β1 has been shown to promote the EMT and EGFR-TKI resistance [16]. More importantly, TGF-β1 overexpression has been correlated to EGFR-TKI resistance and a shorter overall survival in NSCLC patients [43]. We found a strong induction of TGF-β1 expression and secretion in the HCC827 GR cells, but not in the PC9 GR cell lines, suggesting also in this case that the genetic background affects the mechanisms of resistance in NSCLC cells. More importantly, we observed a significant effect of the TGFβR blockade on the migratory ability of HCC827 GR-Low cells. Because TGF-β1 inhibition also partially rescued gefitinib sensitivity in the HCC827 GR cells, these data imply a role of TGF-β1 as a potential target in selected EGFR-TKI-resistant NSCLC cells with elevated levels of expression and secretion of TGF-β1. Several therapeutic strategies for targeting the TGFβ pathway are under investigation [44]. The treatment of NSCLC clones overexpressing TGF-β1 with TGFβ inhibitors might potentially contribute to overcoming resistance to EGFR-TKIs.

Previous studies have demonstrated that acquired resistance to EGFR-TKIs is often multiclonal rather than monoclonal [45]. We might expect that the levels of exposure to EGFR-TKIs might significantly differ within a tumor mass. Indeed, chemotherapeutic agents are conditioned in their penetration through the tumor mass by different factors, including the physical and chemical properties, the inflammatory microenvironment, and the tumor vasculature, resulting in an inadequate and heterogeneous drug concentration in the tumor cells [46]. In this respect, this study provides evidence that such differential drug exposure might lead to the development of different mechanisms of resistance and therefore to an increase in the tumor’s heterogeneity. Because genetic tumor heterogeneity is also likely to occur in advanced NSCLC, an interaction between the genetic background and drug levels might represent an additional source of heterogeneity, as suggested by our findings. In this respect, other mechanisms involved in tumor progression, such as autophagy, which has been also correlated with EMT and TGFβ signaling [47], might be differently regulated in cells exposed to different doses of drugs, contributing to resistance and increasing tumor heterogeneity.

Our results might have potential clinical implications. A better understanding of the different mechanisms of resistance that can coexist in NSCLC cells following differential drug exposure might allow the identification of novel therapeutic targets and the development of therapeutic approaches based on combined or sequential treatments for maximizing their clinical benefits. In this regard, circulating tumor DNA profiling of NSCLC patients might help in detecting mechanisms of resistance and describing intratumor heterogeneity for precision oncology approaches [45,48].

In conclusion, our data demonstrate that molecular mechanisms of resistance to EGFR-TKIs are significantly affected by inadequate drug exposures and the cellular genetic background, and provide the rationale for further studies validating the role of TGF-β1 as a potential target to overcome resistance to EGFR-TKIs and for developing therapeutic strategies aimed at mitigating tumor heterogeneity in NSCLC.

## 4. Materials and Methods

### 4.1. Cell Lines and Reagents

EGFR-mutant (p.E746_A750del) NSCLC cell lines, HCC827 and PC9, were purchased from the American Type Culture Collection (ATCC, Manassas, VA, USA) and from the European Collection of Authenticated Cell Cultures (ECACC, Salisbury, UK), respectively. Cell lines were maintained in a humidified atmosphere at 37 °C and 5% CO_2_ and cultured in RPMI 1640 medium with GlutaMAX supplemented with 10% fetal bovine serum (FBS) (ThermoFisher Scientific, Milan, Italy). Cell lines were authenticated using short-tandem repeat (STR) profiling (Human Cell Line Authentication Service, Eurofins Genomics, Ebersberg, Germany). Gefitinib was purchased from Cayman Chemical (Ann Arbor, MI, USA) and LY2109761 was obtained from MedChemExpress (Monmouth Junction, NJ, USA). All experiments were performed with mycoplasma-free cells.

### 4.2. Generation of Gefitinib-Resistant Cells

HCC827 and PC9 gefitinib-resistant (GR) cell lines were generated using three methods: (1) through continuous exposure to gradually increasing concentrations of gefitinib, starting from 5 nM (for HCC827 cells) or 25 nM (for PC9 cells) to 1 µM over 6 months (GR-High); (2) through continuous exposure to a low fixed dose of gefitinib (10 nM for HCC827 cells and 25 nM for PC9 cells) over 6 months (GR-Low); (3) through intermittent exposure to a low dose of gefitinib (10 nM for HCC827 cells and 25 nM for PC9 cells) for 48–72 h, followed by incubation in gefitinib-free medium for the same time over a period of 6 months (GR-Pulse). The established GR cell lines were routinely maintained in medium containing gefitinib (1 µM for both GR-High cell lines, 10 nM for HCC827 GR-Low and GR-Pulse cells, and 25 nM for PC9 GR-Low and GR-Pulse cells).

### 4.3. Targeted Sequencing Analysis

Comprehensive genomic profiling of the HCC827 and PC9 parental and GR cell lines was performed with the Oncomine Comprehensive Assay v3 (ThermoFisher Scientific). A full list of the genes included in the assay is reported in Table S1. Briefly, libraries were prepared starting from 10 ng of genomic DNA or RNA according to the manufacturer’s instructions. For each sample, the DNA and RNA libraries were combined and clonally amplified on ion sphere particles by emulsion PCR, performed on the Ion One Touch 2 instrument with the Ion PGM template OT2 200 Kit (ThermoFisher Scientific), according to the manufacturer’s instructions. Then, the particles were enriched, loaded on an Ion 318 chip and sequenced on a PGM sequencer with the Ion PGM sequencing 200 kit v2. Raw data were analyzed using the Torrent Suite Software v5.0 (ThermoFisher Scientific). Mutations were identified using the Ion Reporter Software v5.0 with low-stringency settings and verified in the integrative genome viewer from the Broad Institute (http://www.broadinstitute.org/igv/, accessed on 3 March 2023).

### 4.4. RNA-Seq and Bioinformatics Analyses

Total RNA was extracted in duplicates from the GR and parental cell lines using the TRIzol Reagent (ThermoFisher Scientific) and quantified using the Qubit fluorometer with the Qubit RNA HS Assay Kit (ThermoFisher Scientific). Poly-adenylated RNA libraries were prepared using the QIAseq Stranded mRNA Select Kit (Qiagen, Milan, Italy) for each sample and quantified using the QIAseq Library Quant Assay Kit (Qiagen). Paired-end sequencing of the resulting libraries was performed using the High-Output Kit on the NGS NextSeq 500 platform (Illumina, San Diego, CA, USA). The quality of the raw reads was assessed using FastQC on the Galaxy platform (http://galaxyproject.org, accessed on 30 April 2023). The reads (35–151 nt in length) were aligned to the human reference genome (GRCh38/hg38) using HISAT2 with the parameters recommended in the user’s manual [49]. To quantify the number of fragments mapping to the exons for each gene, the software featureCounts v2.0.3 was employed [50] and the genes with total counts lower than 10 across all samples were eliminated. Differential gene expressions between the resistant and parental cells, expressed as log2 fold changes, were obtained with the DESeq2 package v1.32.0, using a threshold for the adjusted *p*-value (Benjamini–Hochberg adjustment for multiple testing) of 0.05. To evaluate the statistical significance of duplicates, dispersions were estimated using the mean values of the log2 fold changes with the Negative Binomial Wald Test included in the DESeq2 package.

Finally, the EnrichR tool [51] (https://maayanlab.cloud/Enrichr/, last accessed on 26 May 2023) was used to perform functional analyses of the enriched pathways included in the Molecular Signatures Database and adjusted *p*-values ≤ 0.0001 were considered statistically significant.

### 4.5. Cell Proliferation Assay

To calculate the IC50 values of gefitinib, the parental and GR cell lines (3 × 10^3^ cells/well) were seeded in 96-well plates. After 24 h, the medium was replaced and the cells were treated with different concentrations of gefitinib for 72 h. Cell proliferation was determined using the tetrazolium-based (MTT) colorimetric assay, as previously described [52]. To evaluate the effects of the combination of gefitinib and LY2109761 on cell proliferation, HCC827 parental and GR cells (3.5 × 10^3^ cells/well) were seeded in 96-well plates in complete medium. The medium was replaced 24 h after seeding and the cells were incubated in medium containing 50 nM gefitinib and/or 5 µM LY2109761 for 72 h. Cell proliferation was determined using the CellTiter 96 AQueous One Solution Cell Proliferation Assay (MTS) (Promega, Milan, Italy), according to the manufacturer’s instructions.

### 4.6. Western Blot Analyses

Whole-protein extracts were analyzed using the Immuno Cruz Western blotting Luminol Reagent (Santa Cruz Biotechnology, Dallas, TX, USA) or the SuperSignal West Pico PLUS Chemiluminescent Substrate (ThermoFisher Scientific). The following antibodies were used: anti-GAPDH, anti-phospho-EGFR (Y1068), anti-phospho-AKT (S473), anti-phospho-p44/42 MAP kinase (T202/Y204), anti-AKT, anti-p44/42 MAP kinase, and anti-Snail (Cell Signaling Technology, Beverly, MA, USA); anti-EGFR, anti-E-cadherin, and anti-N-cadherin (Santa Cruz Biotechnology, Heidelberg, Germany); anti-vimentin antibody (DAKO, Milan, Italy); anti-α-tubulin (Sigma Aldrich, Milan, Italy). Densitometric analysis of the blots was performed using the ImageJ software v.1.8.0. Original blots were shown in Figures S5 and S6.

### 4.7. RNA Isolation and Real-Time PCR

Total RNA was extracted using the TRIzol Reagent and cDNA synthesis was performed with the SuperScript II Reverse Transcriptase (ThermoFisher Scientific) using random hexamers as primers, according to the manufacturer’s protocol. Real-time PCR was performed with a 7900 ABI PRISM instrument (ThermoFisher Scientific) using the SYBR Green qPCR Master Mix (ThermoFisher Scientific). The primer sequences for the investigated genes were as follows: 5′ GCAAATTCCTGCCATTCTGG 3′ (forward) and 5′ CGAAGAAACAGCAAGAGCAGC 3′ (reverse) for E-cadherin; 5′ TCAACTTGCCAGAAAACTCCAG 3′ (forward) and 5′ CCGCAGTGAAAGGTTTTTATCTCT 3′ (reverse) for N-cadherin; 5′ CCCACTCAAAAAGGACACTTCTG 3′ (forward) and 5′ CGTGATGCTGAGAAGTTTCGTT 3′ (reverse) for vimentin; 5′ CCCTGGTTGCTTCAAGGACA 3′ (forward) and 5′ CTGTTGCAGTGAGGGCAAGA 3′ (reverse) for Snail. The primers for human GAPDH were purchased from Qiagen. Relative gene expression levels were calculated using the ΔΔCt method.

### 4.8. Wound-Healing Assay

Cells (1 × 10^6^ cells/well) were plated in 6-well plates in complete medium and serum-starved overnight. Cells were then wounded using sterile pipette tips, washed with PBS, and incubated in cell culture medium containing 2% FBS with or without 5 µM LY2109761. The wounds were photographed at time 0 and after 5 h of incubation. Healing was quantified by measuring the distance between the edges with the ImageJ software. Three different parts of each wound were analyzed and mean values of the distances between the edges were calculated.

### 4.9. Migration Assay

Transwell with 8.0 μm pores polycarbonate membrane inserts were used for cell motility assays (Corning, NY, USA). For the HCC827 cell line, 8 × 10^4^ cells were seeded in the upper chambers in serum-free medium containing 0.1% bovine serum albumin, and medium containing 10% FBS was used as chemoattractant in the lower chambers. The HCC827 cells were allowed to migrate for 24 h. For the PC9 cell line, 3 × 10^4^ cells were seeded in medium containing 2% FBS and allowed to migrate toward 5% FBS for 6 h. Migrated cells were then stained with a crystal violet stain solution (Merck/Millipore, Milan, Italy) after the removal of non-migrated cells using a cotton swab. The crystal violet stain solution was eluted and the absorbance was read at 540 nm.

### 4.10. Conditioned Media Collection and Immunoassay

Cell lines were plated in 24-well plates (2 × 10^4^ and 1.5 × 10^4^ cells/well for HCC827 and PC9 cells, respectively) in complete medium. After 24 h, the cells were washed with PBS and incubated with medium containing 2% FBS. After 72 h, the conditioned media were collected and centrifuged for 10 min at 16,000× *g* to remove cell debris and stored at −80 °C. The TGF-β1 levels were measured in duplicates in conditioned media using the Magnetic Luminex Performance Assay TGF-β base kit (R&D Systems, Minneapolis, MN, USA) and referred to picograms per 10^4^ cells, determined based on the harvesting time.

### 4.11. Immunofluorescence

The parental and GR cells were plated on glass coverslips (3 × 10^4^ cells/well) in 24-well plates. Twenty-four hours after seeding, the cells were permeabilized with 0.1% Triton X-100, blocked in PBS containing 5% bovine serum albumin for 30 min, and incubated overnight with an anti-TGF-β1 antibody (Abcam, Cambridge, UK). An Alexa Fluor 594 secondary antibody (Jackson ImmunoResearch, Ely, UK) was used for signal detection and the nuclei were stained with DAPI. Fluorescence images were captured using AxioPlan2 microscope imaging software Axiovision v.4.8.2.0 (Zeiss, Gottingen, Germany). Original microscopy images were shown in Figure S7.

### 4.12. Statistical Analyses

All statistical analyses were performed with Excel (Microsoft, Redmond, WA, USA). Statistical significance was determined using the two-tailed Student’s *t*-test and *p*-values ≤ 0.05 were considered statistically significant.

## Figures and Tables

**Table 1:** IC50 values for gefitinib in parental and gefitinib-resistant (GR) cell lines.

Cell Line	IC50 ± SEM ^1^
HCC827 parental	13.06 ± 0.98 nM
HCC827 GR-High	>4 µM
HCC827 GR-Low	>4 µM
HCC827 GR-Pulse	>4 µM
PC9 parental	77.26 ± 8.39 nM
PC9 GR-High	>4 µM
PC9 GR-Low	>4 µM
PC9 GR-Pulse	>4 µM

^1^ Values are the mean ± standard error mean (SEM) of three independent experiments.

## Data Availability

The raw RNA sequencing data are available at the following link: https://doi.org/10.5281/zenodo.11057181. The original immunoblot and microscopy images are available in the Supplementary Materials and at the following link: https://doi.org/10.5281/zenodo.11057181. The statistical analysis results are available at the following link: https://doi.org/10.5281/zenodo.11057181.

## Supplementary Materials

The following supporting information can be downloaded at https://www.mdpi.com/article/10.3390/ijms25094844/s1.
