# Supplementary material for: The Impact of Inadequate Exposure to Epidermal Growth Factor Receptor–Tyrosine Kinase Inhibitors on the Development of Resistance in Non-Small-Cell Lung Cancer Cells
Source: Int J Mol Sci. 2024 Apr 29;25(9):4844. doi: 10.3390/ijms25094844 (PMC11084975; doi:10.3390/ijms25094844)
Supplement: Supplementary file 1 [file ijms-25-04844-s001.zip › ijms-2975151-supplementary.pdf]

The Impact of Inadequate Exposure to Epidermal Growth Factor Receptor–Tyrosine Kinase Inhibitors on the Development of Resistance in Non-Small-Cell Lung Cancer Cells

Daniela Frezzetti [1], Vincenza Caridi [1], Laura Marra [1], Rosa Camerlingo [1], Amelia D'Alessio [2], Francesco Russo [3], Serena Dotolo [1], Anna Maria Rachiglio [1], Riziero Esposito Abate [1], Marianna Gallo [1], Monica Rosaria Maiello [1], Alessandro Morabito [4], Nicola Normanno [1,*] and Antonella De Luca [1]

Table of contents

Supplementary Methods……………………………………………………………………..…Page 2

Supplementary Tables………..…………………………………………………………....Pages 3

Supplementary Figures……………….……………………………………….…..……..Pages 4-9

Supplementary Methods

Cell proliferation assay

HCC827 and PC9 parental and gefitinib resistant (GR) cells (2×10^3 cells/well and 6×10^2 cells/well, respectively) were seeded in 96-well plates in gefitinib-free medium. Cell proliferation was determined by tetrazolium-based (MTT) colorimetric assay according to standard procedures.

Supplementary Tables

Table S1: List of genes analysed by targeted sequencing

Hot spot genes	Full-length genes	Copy number genes	Gene fusions
AKT1 AKT2 AKT3 ALK AR ARAF AXL BRAF BTK CBL CCND1 CDK4 CDK6 CHEK2 CSF1R CTNNB1 DDR2 EGFR ERBB2 ERBB3 ERBB4 ERCC2 ESR1 EZH2 FGFR1 FGFR2 FGFR3 FGFR4 FLT3 FOXL2 GATA2 GNA11 GNAQ GNAS H3F3A HIST1H3B HNF1A HRAS IDH1 IDH2 JAK1 JAK2 JAK3 KDR KIT KNSTRN KRAS MAGOH MAP2K1 MAP2K2 MAP2K4 MAPK1 MAX MDM4 MED12 MET MTOR MYC MYCN MYD88 NFE2L2 NRAS NTRK1 NTRK2 NTRK3 PDGFRA PDGFRB PIK3CB PIK3CA PPP2R1A PTPN11 RAC1 RAF1 RET RHEB RHOA ROS1 SF3B1 SMAD4 SMO SPOP SRC STAT3 TERT TOP1 U2AF1 XPO1	ARID1A ATM ATR ATRX BAP1 BRCA1 BRCA2 CDK12 CDKN1B CDKN2A CDKN2B CHEK1 CREBBP FANCA FANCD2 FANCI FBXW7 MLH1 MRE11 MSH6 MSH2 NBN NF1 NF2 NOTCH1 NOTCH2 NOTCH3 PALB2 PIK3R1 PMS2 POLE PTCH1 PTEN RAD50 RAD51 RAD51B RAD51C RAD51D RNF43 RB1 SETD2 SLX4 SMARCA4 SMARCB1 STK11 TP53 TSC1 TSC2	AKT1 AKT2 AKT3 ALK AXL AR BRAF CCND1 CCND2 CCND3 CCNE1 CDK2 CDK4 CDK6 EGFR ERBB2 ESR1 FGF19 FGF3 FGFR1 FGFR2 FGFR3 FGFR4 FLT3 IGF1R KIT KRAS MDM2 MDM4 MET MYC MYCL MYCN NTRK1 NTRK2 NTRK3 PDGFRA PDGFRB PIK3CB PIK3CA PPARG RICTOR TERT	AKT2 ALK AR AXL BRCA1 BRCA2 BRAF CDKN2A EGFR ERBB2 ERBB4 ERG ESR1 ETV1 ETV4 ETV5 FGFR1 FGFR2 FGFR3 FGR FLT3 JAK2 KRAS MDM4 MET MYB MYBL1 NF1 NOTCH1 NOTCH4 NRG1 NTRK1 NTRK2 NTRK3 NUTM1 PDGFRA PDGFRB PIK3CA PRKACA PRKACB PTEN PPARG RAD51B RAF1 RB1 RELA RET ROS1 RSPO2 RSPO3 TERT

Table S2: List of all differentially expressed genes (DEGs) identified in GR cell lines. The content of the table is available as a separate file.

Supplementary Figures

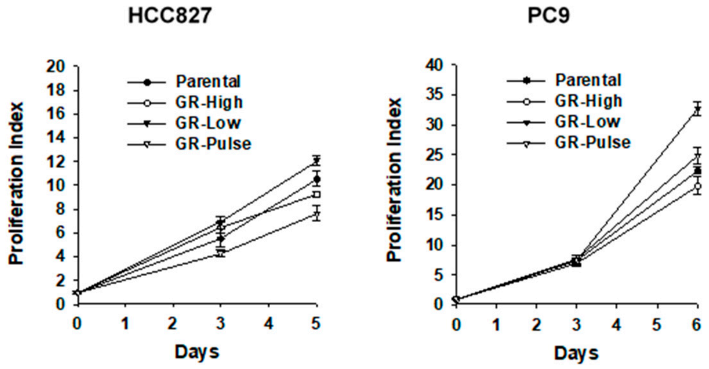

Figure S1: Cell proliferation of gefitinib resistant (GR) NSCLC cells. Parental and GR cells were grown in gefitinib-free medium. Cell proliferation was measured at the indicated time points by MTT assay.

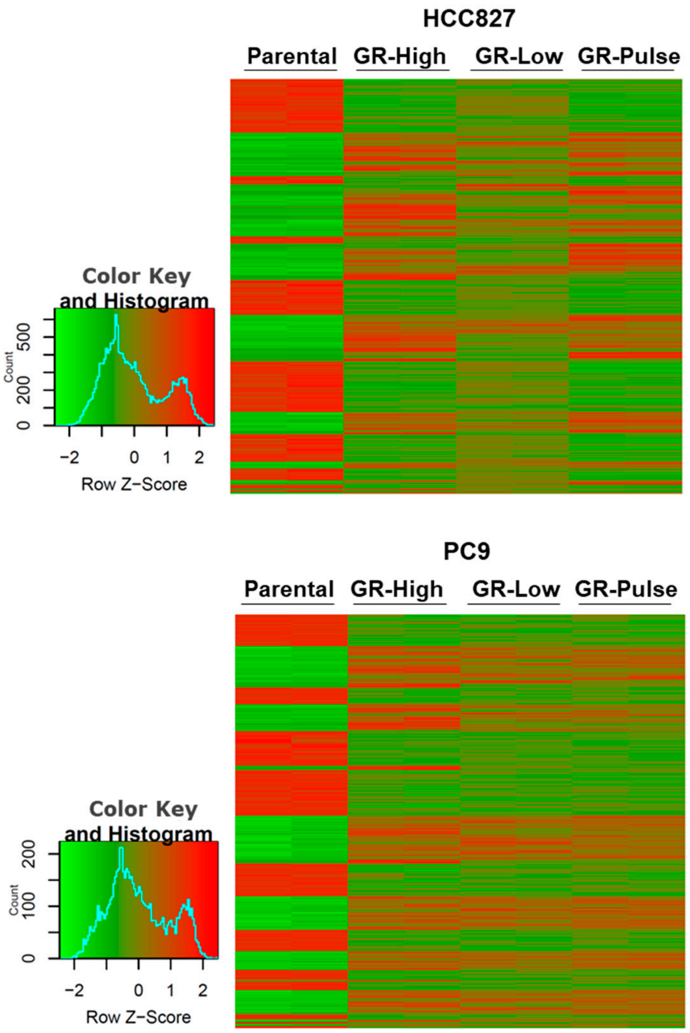

Figure S2: Transcriptome analysis of GR cells.RNA-Seqheatmaps showing genes differentially expressed between GR and relative parental cells. Expression data are shown in duplicate for each cell line.

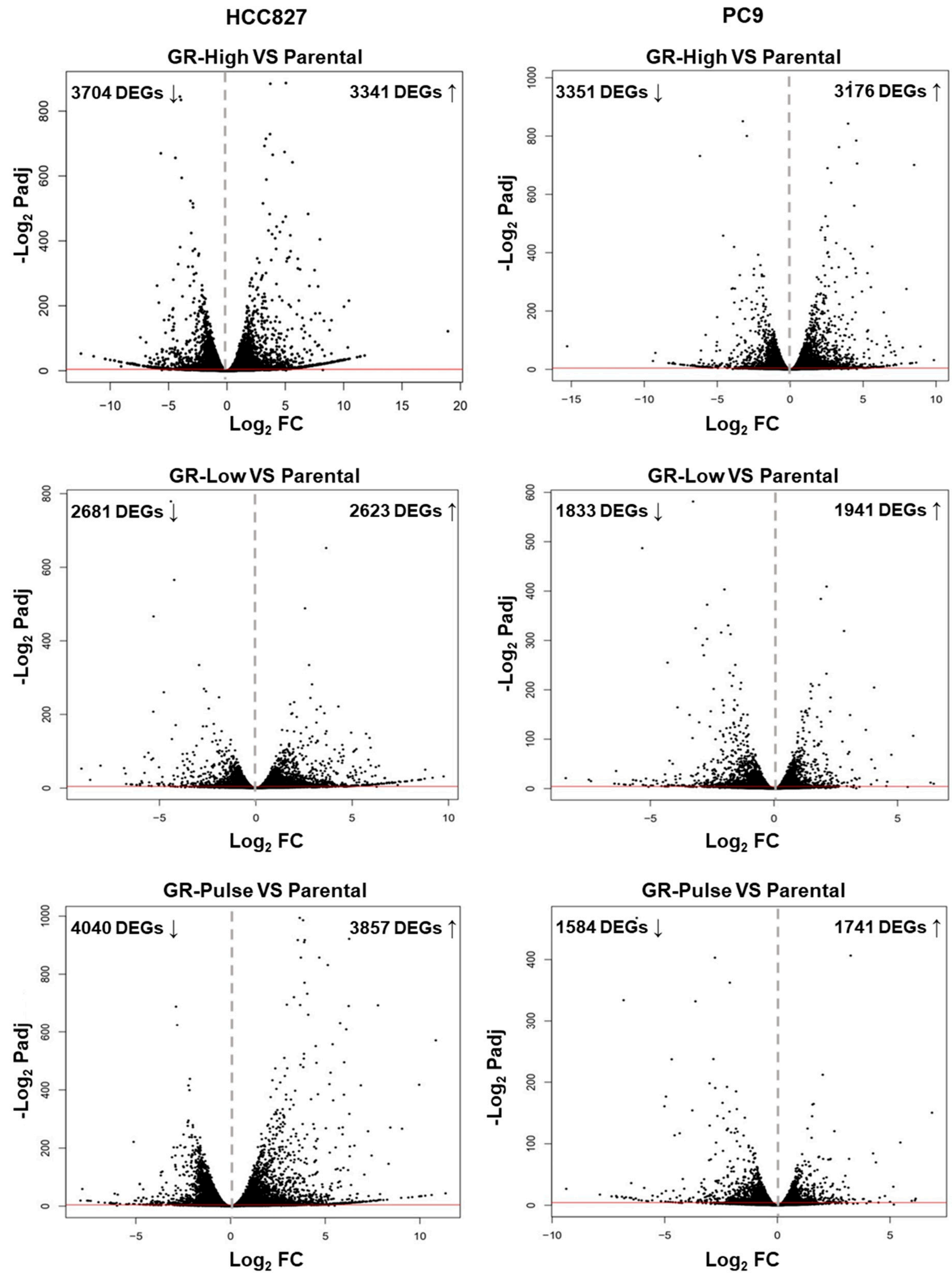

Figure S3: Analysis of differentially expressed genes (DEGs) in GR cells. Volcano plots showing DEGs in GR cells relative to parental cells. The number of statistically significant downregulated and upregulated DEGs is indicated in bold (adjusted p-value < 0.05). Abbreviations: Padj: adjusted p-value; FC: Fold Change.

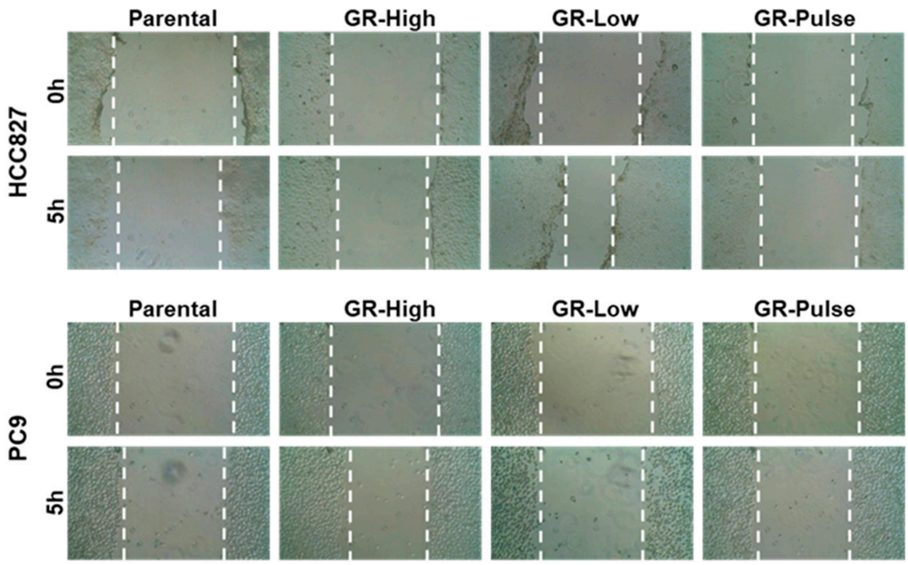

(**a**)

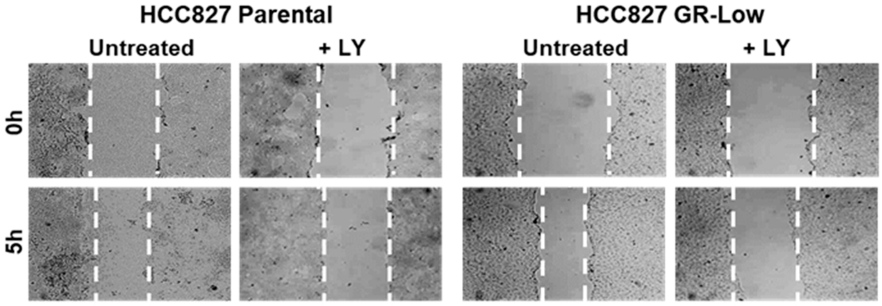

(**b**)

Figure S4: Analysis of migration in GR cells. (**a**) Representative images of wound healing assay at the indicated time points are shown for parental and GR cells. (**b**)Representative images of wound healing assay at the indicated time points performed on HCC827 parental and GR-Low cells, untreated and treated with LY2109761 (LY, 5 μM).

Raw data Figure 2a

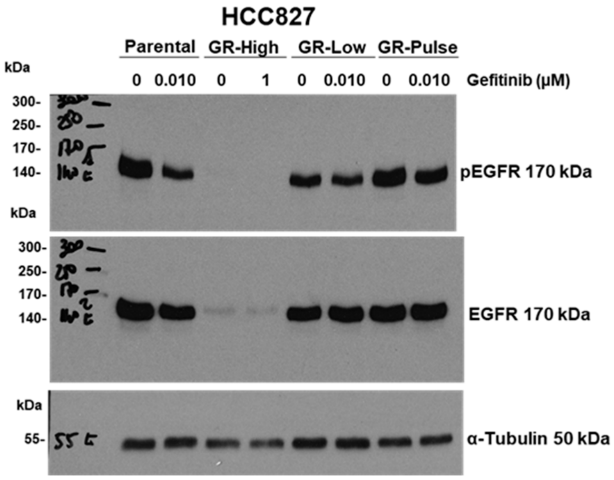

Raw data Figure 2b

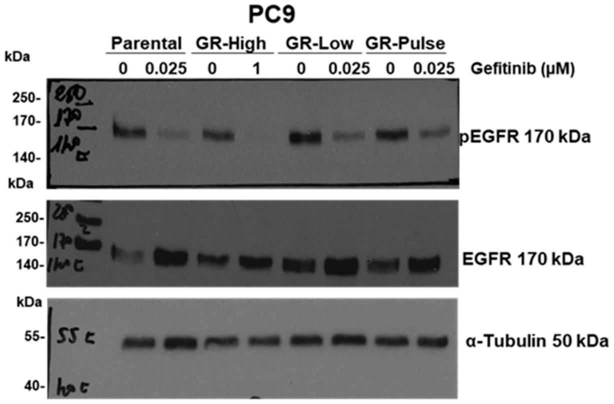

Raw data Figure 2c

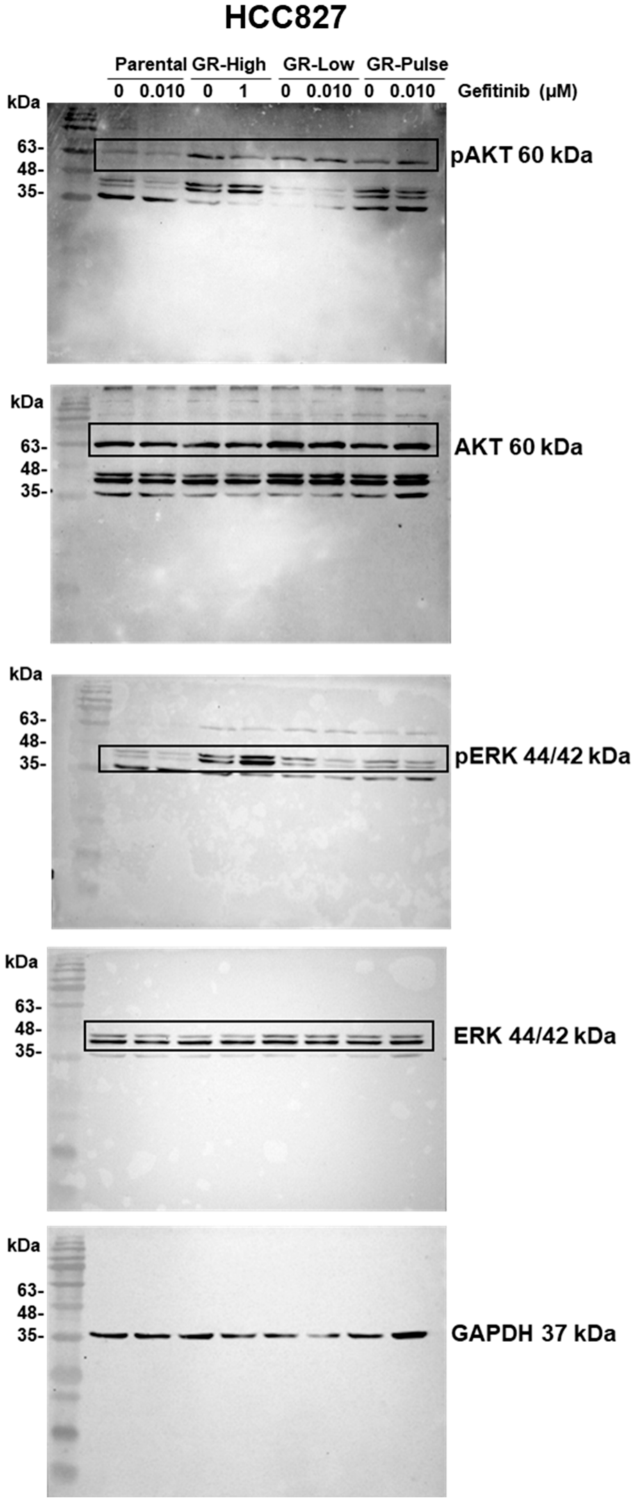

Figure S5. Original immunoblot images of figures 2a, b and c. The original scans of the blots are shown. Squares indicate the images used for final figures.

Raw Data Figure 2d

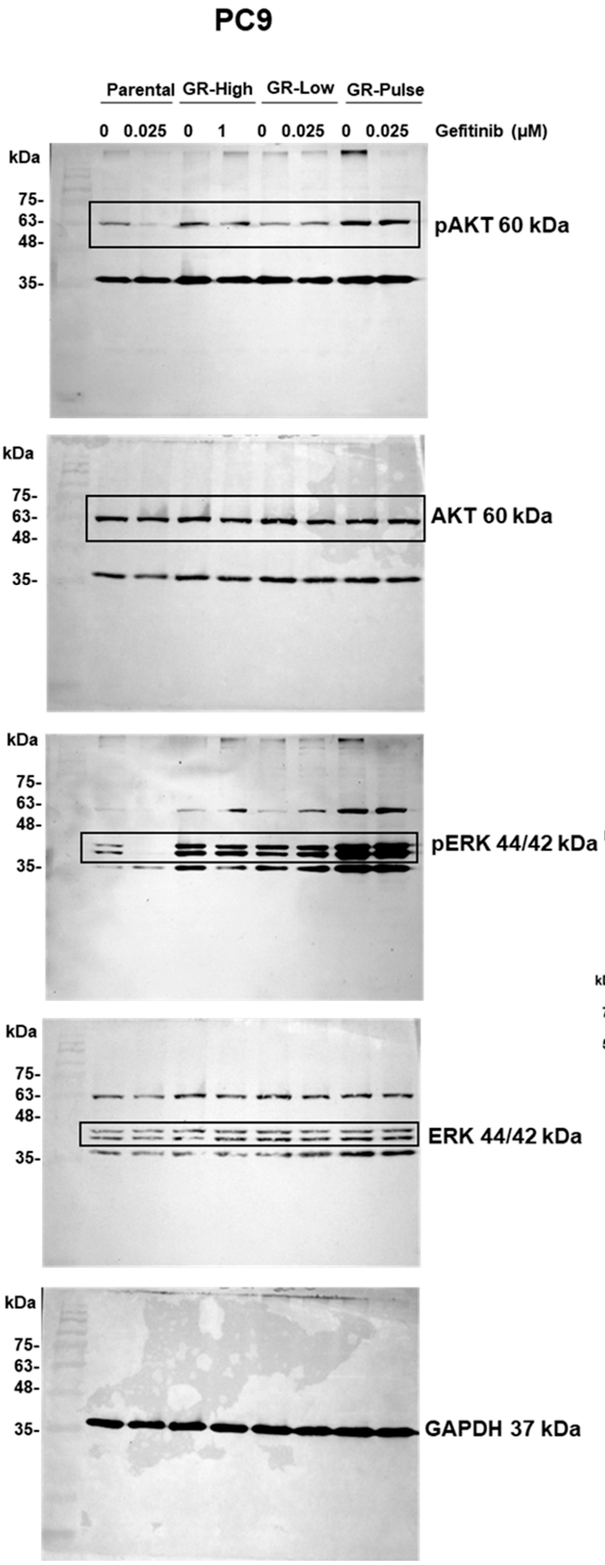

Raw Data Figure 5a

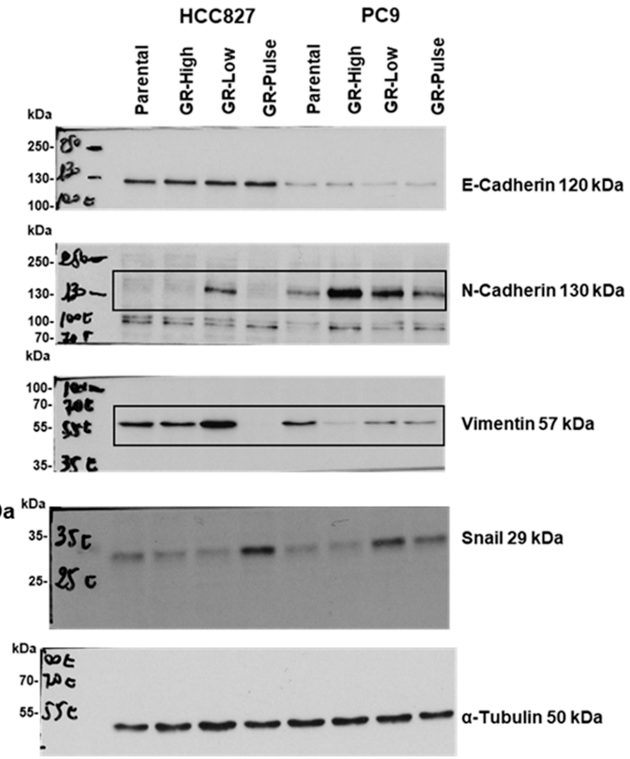

Figure S6. Original immunoblot images of figures 2d and 5a.The original scans of the blots are shown. Squares indicate the images used for final figures.

Raw data Figure 6a

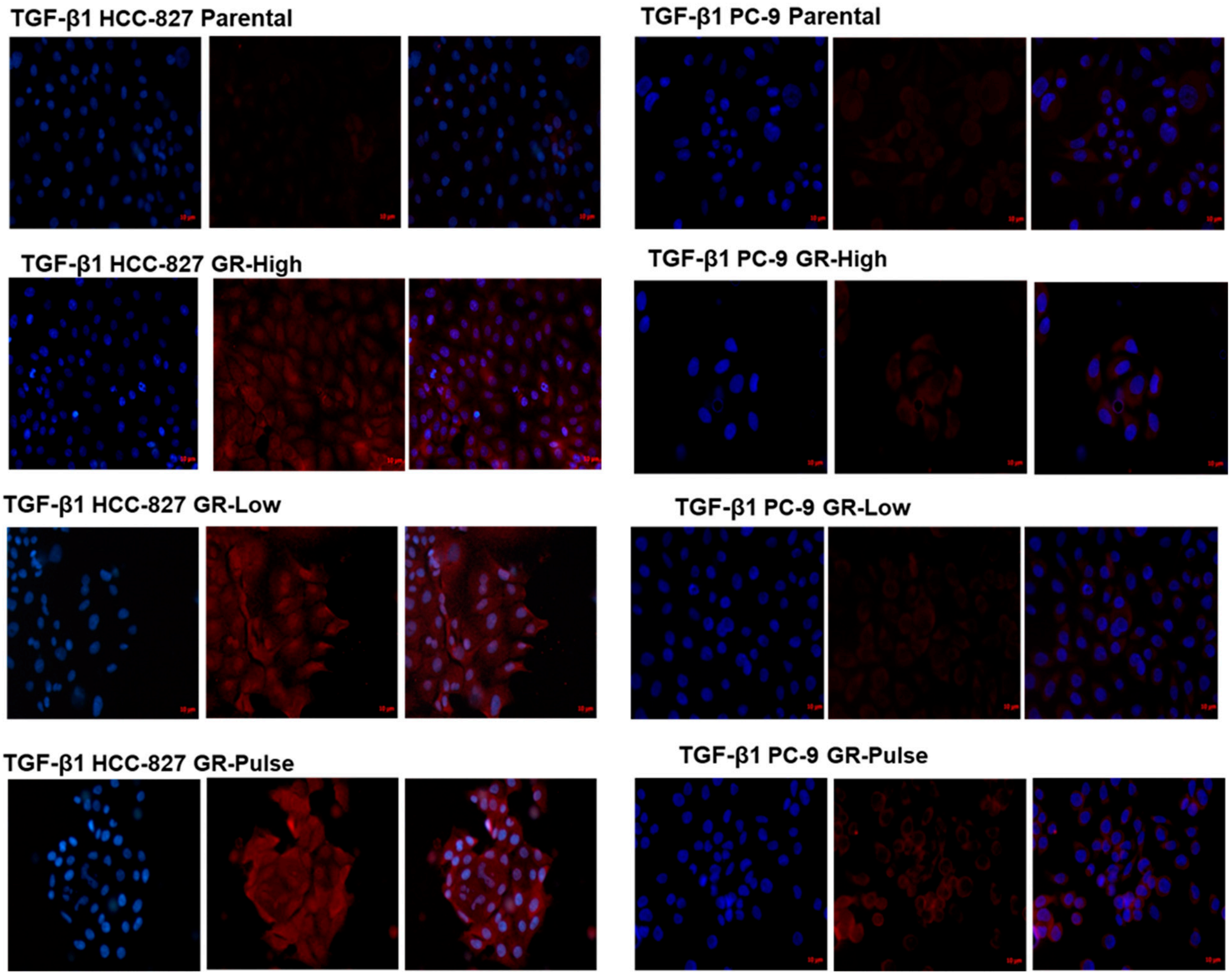

Figure S7. Original microscopy images of figure 6a.The original images obtained with fluorescence microscope are shown.
